# Civil society advocacy in Nigeria: promoting democratic norms or donor demands?

**DOI:** 10.1186/s12914-016-0093-z

**Published:** 2016-07-11

**Authors:** R. Taylor Williamson, Joshua Rodd

**Affiliations:** International Development Group, RTI International, Washington, DC USA; Middlebury College, Middlebury, VT USA

**Keywords:** Advocacy, Civil society, Policy, Nigeria, HIV, Governance, HIV prevention, Rights

## Abstract

**Background:**

Civil society organizations (CSOs) are often assumed to be institutions that facilitate communication between citizens and policymakers. However, CSO advocacy is only as effective as the space allowed by government, the resources available from funders, and their own internal capacity. This article presents findings from a study in Nigeria that explores the advocacy and service delivery roles of CSOs working in Human Immunodeficiency Virus (HIV) prevention and mitigation. We will argue that donor and government treatment of civil society as service delivery organizations, rather than as organizations that participate in democratic norms, have shaped how civil society organizations work to mitigate and prevent HIV.

**Methods:**

From February to April 2012, a team of Health Systems 20/20 staff and one consultant conducted 48 in-depth interviews with civil society organizations, State AIDS Control Agencies (SACAs), donors, international organizations, and networks of people living with HIV to examine a wide range of advocacy efforts by CSOs. For quantitative data collection, sampling frames were assembled from lists of HIV-oriented or involved CSOs. This sampling frame consisted of 2548 CSOs from all 36 states and the Federal Capital Territory. A random sample was then taken from the sampling frame, and we contacted 665 CSOs to arrange interviews. With a response rate of 80.2 %, the project conducted 533 surveys in February 2012.

**Results:**

These surveys showed that CSOs advocacy efforts focused on community mobilization related to behavior change, such as peer education (54.9 % of CSOs) and rallies (58.2 % of CSOs), and not on changing government policies. In-depth interviews highlighted the role of donors and government in shaping a purely apolitical role for most CSOs through funding constraints, regulations, and capacity development choices.

**Conclusions:**

In light of these findings, we present key points for considering the influence of donors and government on civil society advocacy for HIV services and rights. We present evidence that donors, and international organizations, conceive of civil society as apolitical, and not as independent actors that compete for political space. More democratic and rights-based views of civil society’s role, such as holding government accountable for providing services or promoting policy change, are not emphasized.

## Background

Concepts of civil society can be quite varied, but modern interpretations of the term focus on “political space where voluntary associations explicitly seeking to shape the rules that govern…social life” [1]. According to Scholte, these associations can include “academic institutions, business forums, clan and kinship circles, consumer advocates, development cooperation initiatives, environmental movements, ethnic lobbies, foundations, human rights promoters, labour unions, local community groups, relief organisations, peace movements, professional bodies, religious institutions, think tanks, women’s networks, youth associations and more” [1]. Conversely, Gramsci views civil society as part of an “extended state”, by which the ruling class (or the state) is able to maintain hegemony over its citizens [2].

Gaventa synthesizes these two competing ideas – noting that civil society organizations (CSOs) provide a voice for citizens to communicate with government, extend the reach of government by deliver services to citizens, and hold government officials accountable through watchdog efforts [3]. With the Third International Conference on Financing for Development highlighting the role of civil society in “strengthening the mobilization and effective use of domestic resources” for development, CSOs must hold governments accountable for funding and service commitments [4].

When interacting with government, civil society is often dependent on the space allowed by government for civic action. Coston has pointed out that this space falls on a continuum, from collaboration between government and civil society to government repression [5]. Despite the increase in international funding for CSOs to provide HIV services, little research has been done on how donors and governments are influencing CSO advocacy efforts and how CSO advocacy is influenced by donor and government influence. Thus, this article presents the findings from a study in Nigeria that explores the advocacy and service delivery roles of CSOs working in Human Immunodeficiency Virus (HIV) prevention and mitigation.

We will argue that donors and governments treat civil society as service delivery organizations, rather than as organizations that participate in democratic norms, shaping civil society responses to HIV in ways that address donor and government, rather that citizen, needs. Examining this argument now is critical. The scale up of PEPFAR and the Global Fund in the mid-2000s provided CSOs with significant funding to deliver HIV services. As these sources of funding have stabilized over the last 5 years, donors increasingly expect governments to fund a larger percentage of the HIV response. Increased domestic resources for HIV will not be generated, however, without strong civil society advocacy to support appropriate policies and increased funding. Donor and government influence on CSO HIV advocacy, however, is an understudied area of research. The nature of this argument does restrict our analysis to organizations that work on HIV-mitigation and prevention. We cannot generalize our findings to CSOs that do other types of work.

We examine the argument in a number of ways. First, we review the literature on advocacy and state-society relations to develop a theoretical basis for civil society-government engagement and how international donors affect these relationships. Second, we present the findings of mixed methods research on CSO advocacy in Nigeria. A cross-sectional survey highlights the scope and targets of civil society advocacy and civil society’s relationships with government. In-depth interviews are used to understand the role of networks, organizations with a specific advocacy focus, and donor and government influence on the civil society response to HIV in Nigeria. Our article concludes with an analysis of key points for considering the influence of donors and government on civil society advocacy.

## State-society relations and the impact of international donors

According to the Policy Project, advocacy is a set of targeted actions directed at decision makers in support of a specific policy [6]. Advocacy can impact polices at any stage of the policy cycle from agenda setting, formulation, and adoption to implementation and evaluation [7]. Building on this definition, Ezell identifies that a client/agent relationship is necessary; advocacy is conducted by, or on behalf of, a specific group toward decision makers [8]. Edgett contributes a focus on persuasion; noting that advocates attempt to persuade targeted audiences to accept the point of view of the advocate [9]. We can, therefore, think of advocacy as the process of persuading targeted audiences to take a specific policy action, especially government officials. This definition clarifies that the desired outcome should be related to policy change.

The extent to which government defines the purpose and limits of civil society engagement has implications for advocacy. Where civil society is not repressed, state posture can vary from passive support, wherein the state neither supports nor opposes civil society engagement, to active encouragement indicating that the state takes action to engage with civil society [5, 10]. On the passive side, governments do not encourage civil society engagement and citizens must place demands on government without the benefit of state sponsored structures. At the active end of the spectrum, governments conscientiously seek out civil society engagement, and create or promote institutions that require citizen participation [10].

In addition to Blair’s model of state response, Brinkerhoff adds clarity by analyzing the dichotomy between service delivery and advocacy roles of civil society [11]. Government-sanctioned approaches to citizen-state engagement occur when governments actively support civil society engagement, and tend to have a strong focus on CSO service delivery potential [11]. Examples from central Asia and Africa have shown that when governments pre-defined the roles of civil society or business partners, they focus on the potential health service delivery and business investment skills offered by these groups, rather than the potential role of citizen voice [12, 13]. These groups often require strong incentives, monetary or otherwise, to continue their engagement with government [14]. These relationships can be closer to contracting arrangements, as the government decides specific engagement methods and deliverables [14]. Government co-option is a significant concern for CSOs under these arrangements. Brinkerhoff notes that, “As nonprofits have entered into cooperative relations with government, sometimes as contracted service deliverers, they have faced the challenge of balancing the associated benefits with remaining accountable to their primary constituents (i.e., beneficiaries and members)” [11].

Citizen-initiated approaches involve active citizen efforts to reach and influence government, and are critical mechanisms to improve state-society relations, especially within states with passive support for civil society. Coalition-building, citizen scorecards, or media watchdog efforts exemplify demand-side approaches. Civil society initiated engagement with the state tends to have a greater advocacy component under passive regimes, as advocates demand changes to existing state services or the introduction of new programs. Qualitative evidence has shown that active state support for citizen engagement may narrow prescribed avenues, while passive state support allows more options for citizen advocacy [15].

International donors can significantly alter these dynamics by funding programs that prioritize specific roles. For example, a survey in Southern Africa found that 75 % of all funding received by CSOs in 2005 was allocated to program implementation or service delivery, while only 1 % was allocated for advocacy [16]. Seckinelgin found that donors often emphasize quantifiable services, such as condom distribution, HIV counseling and testing, and communication campaigns [17]. As civil society groups respond to available funding for HIV-related services, they shift away from advocacy roles [18]. This dynamic moves CSOs away from being local-level innovators, as donors ask them to implement large-scale service delivery activities with little influence on policy [16].

International donors, through multilateral institutions such as the Global Fund, explicitly attempt to take politics out of health resource allocation decisions [19]. These attempts take a technocratic approach by allocating resources solely through neutral cost-benefit analyses, or what Schedler calls ‘instrumental anti-politics’ [20]. Escobar and Ferguson agree, noting that donors “de-politicize” social problems by providing technical, rather than political, solutions [21, 22]. Within this framing of health as apolitical, there is little room for organizations to tackle challenges from a policy change angle. As a result, donor funding can exclude CSOs from the policy development process, and relegate them to provided apolitical services, pre-determined by international donors and governments [23].

There is considerable disagreement about how CSOs can conduct both advocacy and service delivery roles effectively, or if they need to specialize. Dual roles can exacerbate competing interests; CSOs often receive grants from governments for delivering services, while simultaneously attempting to advocate on behalf of citizens receiving those same services [24]. Brinkerhoff notes that while particular CSOs often do choose either an advocacy or service delivery role, specialization does not preclude the organization from fulfilling multiple roles [14]. Regardless of whether civil society can actually perform dual roles effectively, Jenkins argues that donor emphasis on service delivery excludes organizations that fight for political power, including influencing HIV-related policy frameworks, from receiving funding [23].

Recognizing the challenges inherent to playing both service delivery and advocacy roles, CSOs and people living with HIV can form networks to promote their interests. In theory, these networks focus on advocating for the needs of their members, while leaving service delivery to their members [25]. These networks can be successful; UNAIDS notes that as a result of these networks, the “meaningful involvement of civil society, and particularly of people living with HIV, is now being written into the policies and strategies of many organizations, institutions and AIDS programmes” [26]. Often, however, these networks receive the majority of their funding from international donors and not from membership dues, leading to organizational identity challenges [27]. International donors ask networks to focus on donor priorities, such as coordinating member activities, reporting activities to national AIDS councils, and directing services and training, and not on membership identified actions [28, 29]. As a result, networks are subject to the same de-politicization and co-option pressures that face other CSOs.

## Current policy landscape in Nigeria

In Nigeria, a number of laws, policies, and funding decisions affect PLHIV and HIV programming. For example, the Nigerian government has recently passed a “same-sex” marriage bill, which outlaws Lesbian, Gay, Bisexual or Transgender (LGBT) organizations, harshly penalizes anyone who “performs, witnesses, aids, or abets” a same-sex marriage ceremony, and outlaws any display of a “same-sex amorous relationship” [30]. Additionally, an anti-discrimination and stigma bill was signed in 2012, this law will require monitoring by CSOs to ensure that it is implemented by government actors [31].

In addition to legislation, HIV policies, at the federal, state, and facilities level impact the HIV response. The Nigerian government updates the HIV Policy and National Strategic Plan (NSP) every 5 years; the most recent one ended in 2015 [32]. These documents are opportunities for CSOs to shift the response to the epidemic toward their membership’s needs, including addressing user fees and treatment access. CSO monitoring of the President’s Comprehensive Response Plan for HIV/AIDS is also a critical requirement for ensuring that the NSP is implemented [32].

Funding for HIV is another area of need for advocacy. In 2012, the most recent National AIDS Spending Assessment available, 78 % of HIV funding came from international sources, though this number had fallen from 85 % in 2007 [33]. Increased spending from the Nigerian government, at all levels is necessary to meet financing goals set at the Third International Conference on Financing for Development and improve the sustainability and responsiveness of HIV services.

## Methods and analytical framework

### Analytic objectives

We first examined how frequently Nigerian CSOs, as represented by our survey sample, reported engaging in advocacy work, then examined the advocacy activities CSOs actually carried out. Following this, we sought to identify the characteristics of those CSOs that reported involvement in advocacy work, as compared to those that did not, as well as the characteristics of CSOs involved in self-defined advocacy activities. We also sought information on the landscape and policy environment for CSOs in Nigeria.

### Data collection

From February to April 2012, a team of Health Systems 20/20 staff and one consultant conducted 48 qualitative in-depth interviews with civil society representatives, State AIDS Control Agencies (SACAs), donors, international organizations, and networks of people living with HIV to examine a wide range of advocacy efforts by CSOs. Interviewees were selected through discussions with the National AIDS Control Agency (NACA) and discussions with NACA-led technical working groups.

Data collectors had a set list of questions, but were asked to use their judgment to explore other potential areas of interest. Interview questions focused on CSO service provision, coordination, and HIV-related advocacy, and included perception and process questions. Interviewers took extensive structured notes and used them to develop reports for each interview. Interview notes were taken using pencil and paper and transferred to Word documents. Interviewees were not anonymous in order to identify the perspectives presented in the in-depth interviews.

For quantitative data collection, a sampling frame was assembled from lists of HIV-oriented or involved CSOs that were members of Nigerian HIV civil society networks. Membership in these networks was the sole inclusion criteria for the sampling frame. There were no exclusion criteria. This sampling frame consisted of 2548 CSOs from all 36 states and the Federal Capital Territory. A random sample was then taken from the sampling frame, and 665 CSOs were selected and contacted to arrange interviews. With a response rate of 80.2 %, the project conducted 533 surveys.

The survey requested detailed information on CSO financial, human and infrastructure resources, as well as projects and advocacy activities. In order to understand the scope of an organization’s activities, respondents were asked to report both the total budget for their organization’s HIV activities, as well as budgets for each individual HIV-related project they were currently implementing. On each question about advocacy, interviewers asked the respondents to list their activities, targets, and monitoring mechanisms; those responses were then grouped together under pre-selected categories by the research team.

### Data analysis

All data was entered into Census and Survey Processing System (CSPro), and analysis was carried out using Statistical Package for Social Sciences (SPSS) Version 19. Depending on normality and equality of variances, means testing to identify differences between groups was carried using either Student’s or Welch’s *t*-tests or Mann–Whitney-Wilcoxon Ranked Sum tests.

The authors used Principal Components Analysis (PCA) to create three index variables based on the relationship between six variables (the number of full-time employees, total number of offices, total reported budget, total HIV-related activity budget, total number of part time employees, and total number of volunteers), all related to organizational funding and scale. The three indices captured organization size, organization budget, and organizational access to human resources. The “Size Index” was based on the number of full-time employees, the total number of offices, the total reported budget, and the total HIV-related activity budget and was used in the subsequent analyses.

### Limitations

There are some methodological limitations to our approach. First, because of the relatively small number of organizations in our sampling frame, the results are only valid at the geopolitical zone, not at the state level. Second, we used purposeful sampling to identify key informants, limiting the generalizability of their responses. Finally, we structured the survey to understand the frequency, type, targets, and nature of HIV-focused advocacy. We did not ask questions on specific advocacy “asks”. As a result, specific advocacy issues come from the key informant interviews and mostly reflect national, rather than state or local, issues.

## Results

### Scope of civil society advocacy

We find that 30 % of CSOs include advocacy in their mission statements. CSOs that included advocacy in their mission statements had more staff and funding than those that did not, as indicated by statistically significantly higher scores on the mean Size Index [t(221.5) = −2.781, *p* = 0.006]. Larger organizations are also more likely to have wider ranging mandates and capacities and are more likely to be based in Abuja, the Federal Capital Territory.

**Fig. 1:**
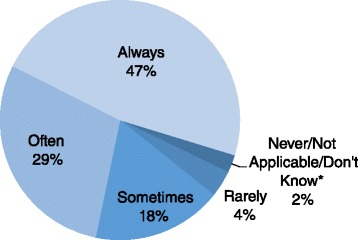
Perceived frequency of participation in advocacy on HIV issues

As Fig. 1 shows, the vast majority of organizations describe themselves as conducting advocacy. When asked to identify their advocacy activities (Table 1), most respondents described activities clearly oriented towards communication, such as peer education (54.9 %) or public rallies (58.2 %). In-depth interviews revealed that most of these activities were focused on community engagement rather than policy change. For example, one network of HIV organizations noted that their members, “advocate to religious leaders and community leaders to reduce stigma and discrimination, create awareness about HIV, mobilize resources for HIV prevention, and provide care and support to PLWHA.” Table 1 does show that nearly 33 % of CSOs reported discussions with elected officials for advocacy purposes.

Advocacy, with a goal of affecting policy change, was rarely cited as a stand-alone activity with a dedicated funding source. Only 14 of the 533 CSOs reported advocacy as a component of an ongoing, funded project. In nine of those 14 CSOs, advocacy was one objective out of several.

**Table 1:** Advocacy activities reported by CSOs

	Number	Percent
Peer education	286	54.9 %
Rallies	309	58.2 %
Discussions w/ elected officials	173	32.6 %
Mass media campaigns	155	29.2 %
Discussions w/ regulators	98	18.5 %
Policy briefs	58	10.9 %
Press releases	53	10.0 %
Press conferences	42	7.9 %
Petitions	23	4.3 %

### Civil society targeting of advocacy activities

Table 2 identifies advocacy targets for CSOs. This table confirms their focus on behavior change rather than influencing government; the most commonly reported targets of advocacy were village leaders and citizens. While it is conceivable that both village leaders and citizens could be targets for mass action to effect policy change, discussions with in-depth interviewees again showed that the CSOs’ actual activities focused on HIV prevention, rather than policy change. Respondents also cited Information, Education, and Communication campaigns, which target village leaders and citizens, as the most common HIV prevention activity. In-depth interviewees rounded out this picture; noting that few CSOs have a scope beyond their local village or community and that they are often disconnected from government, at any level. They noted that the highest scale of operation for most CSOs is a single Nigerian state or city, and that links to networks were weak or non-existent.

**Table 2:** Advocacy targets

	Number	Percent
Village leaders	396	74.6 %
Citizens	249	46.9 %
Elected officials	230	44.1 %
Donors	142	26.7 %
Civil servants	117	22.0 %
Religious leaders	73	13.7 %
Youth & students	28	5.3 %
Women	18	3.4 %

While citizens and village leaders were the most commonly reported targets of advocacy, CSOs also reported directing advocacy efforts towards elected officials (44.1 %). Organizations in our sample reported conducting a median of two such meetings in all of 2011 (Table 3). Only seven percent of CSOs reported meeting with government officials more than once a month (Table 3). In in-depth interviews, civil society respondents noted a lack of contact and coordination with government at all levels. Compared to organizations that did not report targeting government officials, organizations that focused their efforts on elected officials and civil servants were significantly larger, as measured by the Size Index [t(304.2) = −2.203, *p* = 0.028; and t(294.7) = −2.233, *p* = 0.026, respectively]. Five organizations reported meeting with government officials at least once a week; these organizations also reported policy change as a goal of their advocacy work, as reflected in their mission statements.

**Table 3:** Number of one-on-one meetings with governments officials in 2011

Number of meetings	Number	Percent
0	116	22.3 %
1	55	10.6 %
2 to 3	159	30.5 %
4 to 6	94	18.0 %
7 to 12	54	10.4 %
13 to 20	20	3.8 %
21 to 30	11	1.3 %
31 to 50	7	1.0 %
51+	5	1.0 %

Out of 521 total CSO respondents

In in-depth interviews, few CSOs spontaneously named advocacy towards policymakers as a goal. Nonetheless, when asked directly, the majority of CSOs indicated that they would like to influence government policy and decision makers. CSOs, however, rarely devoted financial resources to this goal; advocacy was often pursued in concert with other types of communication strategies. On the other hand, CSOs are generally positive about interacting with government. When asked about challenges to their activities, 43.4 % of CSOs reported that a lack of political support “never” or “rarely” impeded their work. Only 26.5 % reported that they “often” or “always” faced political challenges. CSOs, overall, reported having access to government officials.

### Who are advocacy organizations?

In-depth interviews, conducted with representatives of CSOs that *explicitly* reported advocacy as a core mission, showed that very few CSOs actively advocate for policy change. These organizations fell into one of two broad categories: organizations with explicit advocacy agendas (often supported by foundations, or spun off from international organizations) and networks of CSOs.

Organizations with explicit advocacy agendas included the Planned Parenthood Federation of Nigeria (PPFN), The Initiative for Equal Rights, Journalists against AIDS, and the Health Reform Foundation of Nigeria (HERFON). These organizations often mix advocacy activities with service delivery, depending on available grant funding mostly from private foundations and the Department for International Development (DFID). They also conduct research and analysis to inform specific policy requests. For example, PPFN manages a network of 68 independent clinics, which distribute family planning commodities and information. At the same time, they have directly advocated for changes in Nigeria’s health policy framework, successfully advocating for free antenatal care in four states.

According to key informants, networks, such as the Civil Society HIV/AIDS Network, the Network of People Living with HIV/AIDS in Nigeria, and the Youth Network on HIV/AIDS in Nigeria, are caught between two sets of interests: 1) representing their members and 2) disseminating government funding and policy priorities to their members. Although networks are supposed to give small, local organizations a voice at the federal level, interviewees claimed that these networks are largely co-opted by the international donors and government agencies that fund them. Though some networks do require membership dues, the amounts are nominal. The vast majority of funding for these networks originates with international sources, such as the Global Fund and the World Bank, but is typically managed, either directly or indirectly by NACA [25]. Network interviewees noted that the vast majority of their funding was tied to member coordination and policy dissemination efforts; staff are rarely able to dedicate time to policy analysis and advocacy.

Both advocates and networks have attempted to change some Nigerian federal laws, with the support of NACA. As mentioned in the Introduction, advocates focused on two critical bills the “Same Sex Marriage (Prohibition) Act, 2013” and the “HIV/AIDS Anti-Discrimination Act, 2014”. Interviewees fought to prevent the Same Sex Marriage (Prohibition) Act from passage, while advocating for some changes to and eventual passage of the HIV/AIDS Anti-Discrimination Act. In January 2014, despite advocacy efforts, the same-sex marriage bill was signed into law by President Goodluck Jonathan [30]. Advocates did have some success with the latter bill, as HIV criminalization was removed from the anti-discrimination and stigma bill, and it was signed into law in February 2015 [31].

### Government viewpoints on advocacy

Federal government actors reported holding meetings with civil society to coordinate specific activities or to validate or disseminate specific policies. For example, NACA meets with the networks every 2 months to get feedback on activities funded through Global Fund or World Bank funding that NACA manages. At the end of the fiscal year, they hold work planning sessions to develop networks’ operational plans. NACA does not hold regular meetings to solicit civil society opinions about policy or legislation, though they do hold meetings to disseminate policies on an ad hoc basis. The Federal Ministry of Health reported their main focus is on developing technical guidance and training for service providers, including CSOs. They develop these guidance documents in collaboration with CSOs.

At the state level, SACAs are supposed to coordinate the HIV response through policy development and coordination. In interviews, however, SACA staff expressed frustration at having little to no defined role in monitoring, overseeing, or determining which services civil society provides. In contrast to NACA, which sets policy and controls World Bank and Global Fund money, SACAs have little dedicated funding and their policies and strategic plans are often ignored by CSOs and donors who do not align activities with their priorities. As the President’s Emergency Plan for AIDS Relief (PEPFAR) and Global Fund financing flows entirely outside of SACA structures, CSOs have sources of financing independent from SACAs. Some SACAs have explored providing small grants to CSOs for discrete services, but few do so because they feel the request process is highly bureaucratic and political.

According to interviewees, this coordination gap at the state level impacts policy implementation and monitoring. For example, in one state, CSOs have never provided feedback on HIV policies, nor has the SACA asked for their input. Even in states where CSOs and SACAs do coordinate, CSOs often use reporting tools from their donors which are not aligned to either federal or state guidelines. SACAs do not feel that they are in a position to force compliance, because they cannot provide the community-based services that CSOs can. Some SACA interviewees thought that the federal government preferred that CSOs deliver HIV services instead of state governments, as the federal government retains greater power and influence with weakened states. Some SACA interviewees also thought that CSOs avoid reporting activities in order to hide activities. According to these interviewees, CSOs do not want to be seen as “fully funded”, in case SACAs receive money for CSO grants. We were unable to verify this perception independently. Even though CSOs conduct a great deal of HIV prevention and mitigation work at the state level, SACAs do not think highly of CSOs, claiming that they exist solely because of “the belief that there is a lot of funding available for HIV-related activities”.

### Donor and international organization influence on civil society

In addition to probing civil society and government viewpoints, we also interviewed representatives of organizations that provide most of the funding for civil society’s response to HIV in Nigeria: international organizations and donors. In in-depth interviews, donors emphasized the role of CSOs in extending the reach of HIV-related services, including prevention, testing, care, and treatment, but none mentioned empowering civil society to advocate for the needs of their membership. With the exception of DFID’s support to HERFON, donor support focused on palliative care, orphans and vulnerable children, and HIV prevention reach communities. One interviewee noted that “CSOs have an important role to play because they are able to provide access to ‘hard to reach’ and underserved communities”. International organizations recognized the ability of CSOs to reach areas where the Nigerian state cannot reach, but most thought that Nigerian CSOs did not have strong advocacy skills, as a result of poor coordination with other CSOs.

Interviewees from international organizations noted that while the technical abilities of civil society were quite good, CSOs often had weak organizational and planning capabilities, did not report activities accurately, and did not fully comply with procedures and timelines. Interviewees from donors and international organizations use solutions that homogenize CSO operations, and included mandating minimum service packages, implementing standard financial systems, and specifying program monitoring mechanisms for every CSO that they fund. One interviewee noted that their technical assistance, site visits and budget reviews often ensure that CSOs are “aligned with the donor’s priorities”. Some interviewees suggested that donors, international organizations, and government needed to play a more activist role in “fixing” the civil society response. They suggested further mandates, such as board governance rules, discouraging CSOs from concentrating in major cities, enforcing efficiency standards, and regulating which communities CSOs serve. No international organizations or donors mentioned strengthening the relational or advocacy capacity of CSOs as part of their capacity development efforts.

To promote sustainability, international organizations and donors do try to help CSOs generate internal revenue or appeal to nontraditional funders. They also thought, however, that this work had been unsuccessful, and CSOs would be dependent on international funding to conduct HIV mitigation and prevention activities for the foreseeable future. One interviewee said that “The problem with CSOs is ensuring sustainability because once grants are over, they no longer function.” In addition to being grant dependent, these interviewees thought that CSOs are also grant-responsive. Interviewees from international organizations thought that CSOs are unable to identify organizational priorities, including advocacy. One interviewee noted that, “[CSOs] do not have clearly defined mission, vision or mandate. They compete for anything that is tied to a grant.” This same interviewee thought that many CSOs were simply mechanisms for seeking grants, calling them “Non-Governmental Individuals”.

## Discussion

The priorities and activities of civil society in Nigeria did not develop in a vacuum. They are a response to incentives provided by their membership, government policy, and donors. As is apparent from our in-depth interviews, membership plays a limited role in influencing civil society actions in Nigeria. Federal policy clearly influences CSO priorities, but state directives are often ignored. Most importantly, donors and international organizations, through funding choices and capacity development efforts, also shape civil society advocacy. In this section, we will turn toward analyzing the influences on the civil society response to HIV in Nigeria, identifying areas of potential conflict and co-option.

Throughout this article, we have found evidence that agreed with Rao and Jenkins [18, 23]. Donors, and international organizations, tend to conceive of civil society as apolitical, and not as independent actors that compete for political space. More democratic and rights-based views of civil society’s role, such as holding government accountable for providing services or promoting policy change, are not emphasized. In-depth interviews of donors, as well as current project portfolios, confirmed this characterization. Building on the findings from our cross-sectional survey and in-depth interviews, we propose some key analytical points for understanding the influence of donors and government on HIV-related advocacy.

### International donor actions reduce civil society advocacy potential

As noted by Jenkins, donors often seek out CSOs for purely service delivery roles, shutting off advocacy organizations from funding [23]. They focus on these roles to meet service delivery mandates from their governments, such as the number of people tested for HIV or reached with prevention messages. Nigeria is no different - the vast majority of international funding for HIV toward project implementation and coordination, rather than policy analysis and advocacy [34]. Williamson also estimated that 85 to 90 % of HIV-related funding for CSOs originates from these international donors, providing an incentive for civil society to reorient their work toward their preferences [34].

Internationally funded HIV projects from 2006 to 2012 in Nigeria reflect this service delivery and apolitical framing: the World Bank’s HIV/AIDS Fund (HAF), the Global Fund’s support to CSOs, and various United States Agency for International Development (USAID) and DFID projects primarily focused on service delivery outcomes. Only DFID’s funding of the HERFON stood out as explicitly trying to change federal government policy and legislation.

These funding streams focus the attention of civil society. As CSOs respond to these service delivery grants, they have little incentive to promote the demands of their membership, and their survival becomes aligned with the preferences of donors. A World Bank HAF evaluation found a correlation between CSOs that received grants from HAF and reduced advocacy. For CSOs that did not receive HAF grants, 36 % engaged in advocacy and 16 % held advocacy meetings [35]. At the same time, of those receiving HAF grants, only 9 % engaged in advocacy and 7 % held advocacy meetings [35].

Through the in-depth interviews, we found donor and international NGO perceive that CSO advocacy, financial, and program management skills are fundamentally very weak, even if these organizations do have strong community reach. The 2008 Health Systems Assessment agrees with this evaluation, noting that, “Nigerian CSOs…while vibrant and essentially free to work without constraints, lack the technical grasp, advocacy skills, and networking resources necessary for successful health reform advocacy” [36].

Donor demands for improved service and financial standards, however admirable, have the real effect of homogenizing civil society actions, reducing the space for innovation. While ensuring that CSOs have the capacity to comply with donor regulations, international organizations often require CSOs to use standard technical approaches, financial systems, and monitoring mechanisms. These requirements have the effect of reducing organizational identity and treating CSOs less as independent organizations, and more as service delivery agents of international donors. Even more perniciously, donors and international organizations expressed a desire to more tightly control and regulate civil society actions. These reforms, while unlikely to take place, would further standardize civil society approaches and de-politicize their actions.

### The federal government is complicit in promoting an apolitical view of civil society

In Nigeria, the federal government is an intermediary between donors and CSO-implemented HIV programs, and donors can influence the federal government’s actions toward CSOs. Grants funded by donors, and managed by the federal government, come with clear disbursement requirements, regulations, and compliance mechanisms. By agreeing to these requirements, the Nigerian government implicitly agrees that CSOs working to mitigate HIV are apolitical actors. The federal government, of course, has every reason to accept this framing.

Instead of empowering CSOs to advocate for increased state responses to the HIV epidemic, they can co-opt CSOs into service delivery roles that extend the reach of government to communities without services. This intermediary role also allows the federal government to retain control over funding, further strengthening the power of the state vis-à-vis civil society. As a result, the incentives of the federal government and donors are often aligned. The federal government is acting in support of international donor goals: disbursing service delivery grants and monitoring CSO progress. When CSOs do get involved in policy, they participate in government-directed, and donor-demanded processes, such as HIV strategy or policy development.

### Civil society service delivery weakens state governments

Unlike the federal government, state governments have little to no defined role in monitoring, overseeing, or determining which services civil society provides. SACAs have little to no role in the grant disbursement process or independent funding, and CSOs see little need to report to, or coordinate activities with, them. State governments are often bypassed by the federal government, who coordinates directly with CSOs. As a result, even when states have specific HIV strategies or policies, state officials have little influence on HIV prevention and mitigation activities.

### Larger CSOs were more likely to have better developed advocacy mechanisms

Though our study did not look directly at unrestricted funding, larger organizations do have a wider variety of funding sources, greater potential for generating overhead, and, therefore, more resources to conduct advocacy. Of course, resources are not the only metric to consider. At its core, technical capacity to analyze policy or engage with the government, is a product of institutional decisions about how to allocate resources. CSOs must also see institutional value in allocating scarce resources to advocacy efforts. A few do see this value at the federal level, where a number of advocacy organizations are focused, but they also bypass state governments as irrelevant to their work.

### CSOs respond to donor and government influence by focusing efforts at the local level

Consistent with the donor emphasis on service delivery, CSOs reported focusing their advocacy efforts on village leaders and the community to influence behavior change. This focus on influencing village leaders is a rational response to both donor preferences and the limited community-level reach of the Nigerian government. As a result, most CSOs have little contact with government at any level. While they are often members of networks that ostensibly advocate for their interests at the federal level, these networks, as has been shown earlier, actually take direction from the federal government.

## Conclusions

Over the last decade, billions of dollars have flowed through civil society to prevent and mitigate the effects of HIV. Comparatively little funding has been available to support civil society to challenge repressive policies, advocate for better government services, or hold government accountable for upholding their obligations in the response to HIV. With new emphasis on raising domestic resources to prevent and mitigate HIV, the era of abundant international funding is over. CSOs will have to learn how to hold governments accountable for meeting the commitments to which they have agreed, in order to maintain the momentum that has been built over the last decade. Donor and government actions toward CSOs, which have mostly accommodated an apolitical and service delivery focus, will also have to change to ensure that governments lead HIV mitigation and prevention efforts.

## Abbreviations

AIDS, Acquired Immunodeficiency Syndrome; CSO, Civil Society Organization; CSPro, Census and Survey Processing System; DFID, Department for International Development; HAF, HIV/AIDS Fund; HERFON, Health Reform Foundation of Nigeria; HIV, Human Immunodeficiency Virus; NACA, National Agency for the Control of AIDS; PCA, Principal Components Analysis; PEPFAR, Presidents Emergency Plan for AIDS Relief; PPFN, Planned Parenthood Foundation of Nigeria; SACA, State AIDS Control Agency; SPSS, Statistical Package for the Social Sciences; UNAIDS, Joint United Nations Programme on HIV/AIDS; USAID, United States Agency for International Development
